# Healthcare Utilization and Economic Burden of Pediatric Lower Respiratory Tract Infections Across Five Tertiary Hospitals in Saudi Arabia

**DOI:** 10.3390/pediatric18030071

**Published:** 2026-05-25

**Authors:** Nawaf M. Almuqati, Mohammed Y. Al-Hindi, Hibah A. Moussa, Sama H. Alzahrani, Manar A. Almuntashri, Mansour A. Al-Qurashi, Mawyah O. Barayyan, Shaykhah M. Bin-Sifran

**Affiliations:** 1 College of Medicine, King Saud bin Abdulaziz University for Health Sciences, Jeddah 9515, Saudi Arabia; almuqati20237@ksau-hs.edu.sa (N.M.A.); almuntashri20146@ksau-hs.edu.sa (M.A.A.); qurashima@ngha.med.sa (M.A.A.-Q.); 2 Department of Neonatal Intensive Care, King Abdulaziz Medical City—Western Region, Ministry of National Guard Health Affairs (MNGHA), Jeddah 9515, Saudi Arabia; 3 King Abdullah International Medical Research Centre (KAIMRC), King Abdulaziz Medical City, Ministry of National Guard Health Affairs (MNGHA), Jeddah 9515, Saudi Arabia; 4 College of Medicine, King Abdulaziz University, Jeddah 80200, Saudi Arabia; hmousa0004@stu.kau.edu.sa (H.A.M.); salzahrani1615@stu.kau.edu.sa (S.H.A.); 5 School of Medicine and Dentistry, University of Central Lancashire, Preston PR1 2HE, UK; mombarayyan@lancashire.ac.uk; 6 Ibn Sina National College for Medical Studies, Jeddah 21462, Saudi Arabia; shiekhahbinsifran@hotmail.com

**Keywords:** epidemiology, healthcare costs, intensive care unit, lower respiratory tract infections, pediatric, Saudi Arabia

## Abstract

Objectives: We aimed to describe the healthcare utilization and economic burden of lower respiratory tract infections (LRTIs) among children aged 1–24 months across five tertiary hospitals in Saudi Arabia. Methods: This multicenter retrospective cohort study included 14,320 children diagnosed with LRTIs between August 2021 and July 2025. Data were extracted from the electronic medical records of the Ministry of National Guard Health Affairs. Demographics were analyzed using a patient-level dataset, whereas healthcare utilization and costs were evaluated at the episode level. Data were analyzed using descriptive and inferential statistics and multivariable logistic regression. Results: A total of 14,320 children contributed 22,895 LRTI-related episodes during the study period. Nearly half of the cohort (49.4%) were aged 1–6 months, and bronchiolitis was the predominant diagnosis (84.6%), followed by pneumonia (15.1%). Overall, 34.4% of patients required hospitalization, while 7.1% required ICU admission. LRTIs accounted for 21.0% of all pediatric ward admissions across participating hospitals. Total direct healthcare costs reached USD 23.0 million. Although ICU admissions represented only 7.1% of episodes, they accounted for 45.1% of total healthcare expenditures. In multivariable analysis, pneumonia was independently associated with higher odds of ICU admission compared with bronchiolitis (aOR 2.91, 95% CI 2.43–3.48; *p* < 0.001). Significant seasonal variation in LRTI episodes was observed, with higher episode volumes during winter months (*p* = 0.004). Conclusions: Pediatric LRTIs impose substantial clinical and financial burdens, particularly among younger infants, marked by disproportionate ICU-related costs.

## 1. Introduction

Globally, lower respiratory tract infections (LRTIs) are a major cause of morbidity and mortality, ranking as the third leading cause of death among children under five years of age [1]. Worldwide, the incidence of LRTIs is approximately 12,197.8 new cases per 100,000 children under five years of age [2]. LRTIs affect the respiratory tract below the larynx and encompass conditions such as bronchiolitis, bronchitis, tracheitis, and bronchopneumonia [3]. Common risk factors for LRTIs include indoor air pollution, poor nutrition, low socioeconomic status, insufficient vaccination, and preterm delivery [4].

In Saudi Arabia, LRTIs represent a major cause of morbidity and hospital admissions among infants and young children. National guidelines from the Saudi Initiative of Bronchiolitis Diagnosis, Management, and Prevention identify bronchiolitis as one of the most common causes of hospitalization among children under two years of age [5].

Regional studies demonstrate that bronchiolitis and pneumonia are the most frequent clinical presentations of LRTIs, particularly among infants, who experience higher hospital admission rates, greater disease severity, and increased utilization of intensive care services [5,6]. Hospital-based studies and national consensus documents in Saudi Arabia report that respiratory syncytial virus (RSV) accounts for 25% to over 75% of bronchiolitis cases, particularly among younger infants. This wide prevalence range is attributed to differences in diagnostic approaches, patient populations, healthcare settings, and seasonal timing [7]. Evidence suggests that LRTIs display a clear seasonal pattern; although viral detections occur year-round, they increase markedly during the winter months, particularly between December and March [8].

Despite the recognized burden and seasonal patterns of LRTIs in Saudi Arabia, comprehensive multicenter epidemiological data regarding patient demographics, clinical outcomes, and resource utilization for children admitted with LRTIs remain limited. Existing studies are predominantly single-center and may fail to capture broader regional variations in disease burden, seasonal trends, and healthcare utilization across diverse hospital settings. This nationwide evidence gap constrains efforts to optimize clinical management and inform healthcare planning [6,9].

Understanding the epidemiology of LRTIs is critical for guiding resource allocation, advancing care pathways, and shaping public health interventions in a setting with diverse demographic and climatic influences. Moreover, reliable baseline data on disease burden and healthcare utilization are essential to evaluate the impact of new preventive strategies and inform health policy decisions. Therefore, this study aimed to describe the healthcare utilization and economic burden of LRTIs among children aged 1–24 months across five tertiary hospitals in Saudi Arabia.

## 2. Methodology

### 2.1. Study Design and Setting

This multicenter retrospective cohort study was conducted across facilities of the Ministry of National Guard Health Affairs (MNGHA) in Saudi Arabia between August 2021 and July 2025. The study included five tertiary care hospitals: King Abdulaziz Medical City–Riyadh (KAMC-R), King Abdulaziz Medical City–Jeddah (KAMC-J), King Abdulaziz Hospital (KAH), Imam Abdulrahman Al Faisal Hospital (IABFH), and Prince Mohammed Bin Abdulaziz Hospital (PMBAH).

### 2.2. Study Population

The study population comprised children aged 1 to 24 months who were diagnosed with LRTIs and admitted to the pediatric ward, the intensive care unit (ICU), or the emergency department. LRTIs were defined using predefined ICD-10 diagnostic codes (J12–J18 and J20–J22) recorded in the electronic medical record during routine clinical care. Because this was a retrospective administrative database study, standardized prospective clinical criteria definitions were not applied at the time of data collection.

### 2.3. Inclusion and Exclusion Criteria

Patients were selected using consecutive sampling; all eligible patients presenting during the study period were included. The inclusion criteria comprised children aged 1 to 24 months with an LRTI diagnosis.

### 2.4. Data Collection

Data were extracted from three primary sources using a standardized Microsoft Excel spreadsheet. First, the Central Data Management department provided demographic and clinical variables, including age, sex, admission and discharge dates, and primary diagnoses. Second, an internal data analytics portal was utilized to obtain aggregated statistics on total admissions to inpatient (IP) services, the ICU, and the ED. Third, the Patient Business Center provided standardized cost data.

Cost estimates were derived from standardized institutional rates and reflect an administrative cost model rather than patient-level expenditure data. General pediatric ward admission costs per patient-day included the following: a daily consultant visit (Most Responsible Physician [MRP]), USD 53; hospital care in a shared room, USD 133; nursing care, USD 27; and basic medical supplies, USD 13. Pediatric ICU admission costs per patient-day included the following: hospital care in the neonatal/pediatric ICU, USD 400; NICU critical care consultation, USD 267; daily consultant visit (MRP), USD 53; nursing care, USD 80; and basic medical supplies, USD 67. The direct ED cost was USD 80 per visit.

### 2.5. Sample Size and Study Instrument

A total of 14,320 children aged ≤24 months with LRTIs were included in the study. A nonprobability consecutive sampling method was employed. Patients were identified using standardized International Classification of Diseases, Tenth Revision (ICD-10) codes J12–J18 and J20–J22. A complete list of included codes is provided in Supplementary Table S1. The study instrument was a structured data extraction sheet capturing demographic, clinical, and cost variables. Extracted data included patient identification numbers, age, sex, date of birth, admission and discharge dates, diagnosis, in-hospital mortality during the index admission, overall length of stay (LOS), ED involvement, ICU LOS, total costs, and additional remarks.

## 3. Episode Definition and Analytic Datasets

Episodes were defined administratively based on the timing of records; ED visits followed by ward or ICU admission within 24 h were consolidated into a single clinical episode. This approach was used to reflect one continuous LRTI-related healthcare event rather than multiple administrative care settings within the same presentation. The 24 h threshold was selected to distinguish continuation of the same acute clinical encounter from separate healthcare visits.

**Figure 1:**
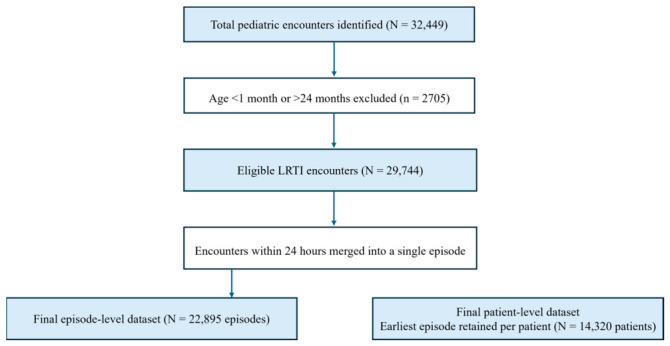
Flow diagram of pediatric LRTI encounter selection and dataset construction.

Therefore, two analytic datasets were constructed to serve distinct methodological purposes. The patient-level dataset included only the earliest LRTI episode for each patient, whereas the episode-level dataset included all eligible clinical episodes after application of the 24 h episode-construction rule, and was used to evaluate healthcare utilization, LOS, costs, and seasonal trends (Figure 1). This episode-construction approach may have reduced the absolute number of recorded ED encounters because ED visits followed by hospitalization within 24 h were treated as part of the same clinical episode.

### 3.1. Potential Bias and Measures to Minimize Bias

To reduce selection bias and ensure a representative sample of the MNGHA population, consecutive sampling was employed across the five tertiary hospitals. To minimize information bias and ensure consistent case identification, patients were identified using standardized ICD-10 codes extracted directly from electronic medical records rather than relying on subjective clinical recall. Furthermore, data extraction was conducted using a structured spreadsheet to standardize variable collection and mitigate transcription errors.

### 3.2. Statistical Analysis

Data were analyzed using IBM SPSS Statistics for Windows, Version 26.0 (IBM Corp., Armonk, NY, USA). Categorical variables were summarized as frequencies and percentages, whereas continuous variables were presented as medians and interquartile ranges (IQRs) or means ± standard deviations, as appropriate for the data distribution. Comparisons of continuous variables were performed using nonparametric tests owing to the non-normal distribution of the data. Associations between categorical variables were assessed using the chi-square test, or the Fisher exact test when expected cell counts were small. The Mann–Whitney U test and Kruskal–Wallis H test were used for comparisons of non-normally distributed continuous variables between two groups and more than two groups, respectively. Multivariable logistic regression analysis was performed using the patient-level dataset to evaluate factors associated with ICU admission. To assess seasonality, a Kruskal–Wallis H test was conducted to compare the median number of monthly episodes across the 12-month calendar cycle over the three-year 2022–2024 period. Sensitivity analyses were performed by excluding the upper 1% of length-of-stay (LOS) observations to evaluate the influence of extreme prolonged hospitalizations on overall cost estimates. All statistical tests were two-tailed, and a *p*-value < 0.05 was considered statistically significant.

## 4. Ethical Considerations

The study was approved by the Institutional Review Board (IRB) of the King Abdullah International Medical Research Center (KAIMRC) (protocol number: NRJ25/060/7). A waiver of informed consent was granted because of the retrospective design of the study and the exclusive use of de-identified patient data, posing minimal risk to participants.

## 5. Results

### 5.1. General Description of the Population

Across the five hospitals, 14,320 children aged 1 to 24 months with LRTIs were included in the patient-level dataset. Of these, 8099 (56.6%) were male, and 6219 (43.4%) were female. The overall mean age was 8.19 months (median, 7 months; IQR, 4–12), with the largest proportion falling within the 1- to 6-month age cohort (*n* = 7079; 49.4%) (Table 1). Overall, 9401 patients (65.6%) were managed exclusively in the ED, whereas 4919 (34.4%) required hospital admission. Among the admitted patients, 1010 (7.1% of the total cohort) required ICU admission. The overall mortality rate was 0.08% (*n* = 11), with all deaths occurring among younger infants. Acute bronchiolitis was the predominant diagnosis (84.6%), followed by pneumonia (15.1%) and other indications (0.3%).

### 5.2. Age-Related Differences in Outcomes

Diagnostic distribution varied significantly by age (*p* < 0.001) (Table 2). Children aged 1 to 6 months exhibited the highest prevalence of acute bronchiolitis (*n* = 6631; 46.4% of all bronchiolitis cases); this prevalence steadily decreased with advancing age. Similarly, age was significantly associated with ICU admission rates (*p* < 0.001). Most ICU admissions occurred among patients aged 1–6 months (*n* = 717; 71.0%), followed by those aged 7–12 months (*n* = 163; 16.1%), 13–18 months (*n* = 73; 7.2%), and 19–24 months (*n* = 57; 5.6%).

### 5.3. Factors Associated with ICU Admission

The results of the univariate and multivariable logistic regression analyses for factors associated with ICU admission are summarized in Table 3. In the multivariable model, increasing age was associated with significantly lower odds of ICU admission (aOR 0.88, 95% CI 0.87–0.89, *p* < 0.001). Patients diagnosed with pneumonia had nearly three times higher odds of ICU admission compared to those with acute bronchiolitis (aOR 2.91, 95% CI 2.43–3.48, *p* < 0.001). Regarding inter-hospital variation, only KAH remained significantly associated with higher admission odds relative to the reference site, IABFH (aOR 2.14, 95% CI 1.64–2.79, *p* < 0.001). Male sex showed no significant association with ICU admission status in either the univariate or multivariable models.

### 5.4. Resource Utilization

During the study period, 22,895 LRTI episodes resulted in an aggregate direct healthcare expenditure of USD 22,957,719 (Table 4). Ward-related expenses totaled USD 10,765,986, whereas ICU-related expenses totaled USD 10,360,133. Additionally, USD 1,831,600 was attributed to ED costs. Total direct costs of USD 22,957,719, therefore, comprised hospitalized episode costs of USD 21,126,120 (Table 5) and ED visit costs of USD 1,831,600. Despite representing only 7.1% of the patients, ICU care accounted for 45.1% of total healthcare expenditures. The median cost per episode was USD 907 (IQR, 680–1586) in the ward and USD 4333 (IQR, 2600–8665) in the ICU. The median LOS was 4 days (IQR, 3–7) in the pediatric ward and 5 days (IQR, 3–10) in the ICU. The episode-level dataset identified 1166 ICU episodes, compared to 1010 unique patients with at least one ICU admission in the patient-level dataset, reflecting patients with more than one ICU episode during the study period. Approximately 65 hospitalization episodes were identified as extreme LOS outliers and primarily reflected medically complex patients requiring prolonged ICU-level care, including extended ventilatory support in some cases. Sensitivity analysis excluding the upper 1% of LOS observations reduced the total estimated costs from approximately USD 23.0 million to USD 16.9 million. However, median costs and interquartile ranges remained largely unchanged, and the observed cost differences in both diagnosis-based and hospital-stratified analyses remained statistically significant after exclusion of extreme LOS values.

Among hospitalization episodes, 5106 (77.1%) episodes were attributed to acute bronchiolitis and 1500 (22.6%) to pneumonia, with the remaining 20 episodes (0.3%) attributed to other LRTI diagnoses. Total hospitalization costs were USD 12,517,133 for acute bronchiolitis and USD 8,571,493 for pneumonia, representing 59.2% and 40.6% of total costs, respectively. Median episode costs were higher for pneumonia than for acute bronchiolitis (USD 1020 [IQR: 680–2493] vs. USD 907 [IQR: 453–1587], *p* < 0.001).

Costs per hospitalization episode varied significantly across hospitals (Kruskal–Wallis H(4) = 238.92, *p* < 0.001) (Table 5). Bonferroni-adjusted post hoc pairwise comparisons revealed statistically significant pairwise differences among most hospital pairs, with no significant differences observed between KAH and KAMC-J or between PMBAH and IABFH.

To evaluate the overall burden of disease across the participating sites, LRTI-related admissions were aggregated and compared against total all-cause pediatric hospitalizations from all five centers. In 2022, there were 1370 hospital ward admissions for LRTI, while 4732 were admitted for other reasons. This means that LRTI accounted for 22.5% of all admissions. There were 1505 LRTI-related admissions and 5005 non-LRTI admissions in 2023, which is 23.1% of all admissions. In 2024, 1413 LRTI-related admissions occurred compared with 5154 non-LRTI admissions, accounting for 21.5% of total admissions. Overall, LRTIs accounted for 21.0% of all pediatric ward admissions across participating hospitals during the study period. The highest admission proportion was observed at KAMC-R (28.2%), followed by KAH (19.3%), PMBAH (15.4%), KAMC-J (15.2%), and IABFH (13.9%).

### 5.5. Seasonality

**Figure 2:**
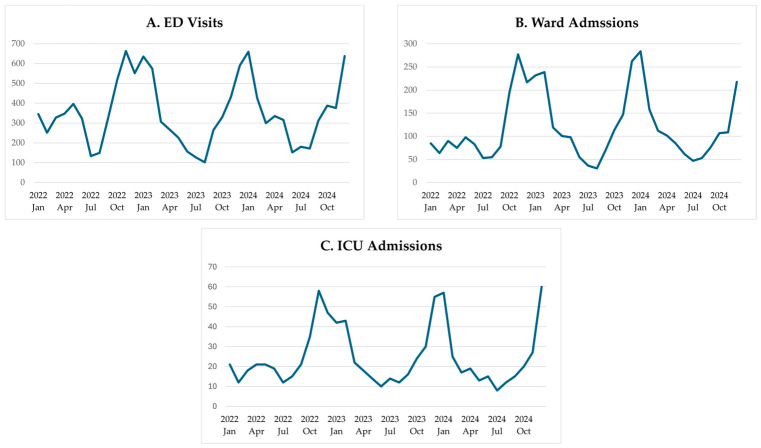
Monthly trends in pediatric lower respiratory tract infection (LRTI)-related ED visits, ward admissions, and ICU admissions from 2022 to 2024.

A statistically significant seasonal variation in LRTI episodes was observed χ^2^(11) = 27.764, (*p* = 0.004). Higher episode frequencies were observed during winter months (Figure 2).

## 6. Discussion

This multicenter cohort study demonstrated that LRTIs represent a substantial clinical and economic burden across tertiary hospitals in Saudi Arabia. LRTIs accounted for 21.0% of all pediatric ward admissions; overall, 34.4% of patients required ward hospitalization, and 7.1% required ICU admission. Although ICU admissions represented a small proportion of total episodes, they accounted for 45.1% of total direct healthcare costs, underscoring the disproportionate financial impact of severe disease. Across the 22,895 recorded episodes, total direct costs were USD 23.0 million. Bronchiolitis was the predominant diagnosis (84.6%), followed by pneumonia (15.1%). Multivariable analysis further demonstrated that pneumonia was independently associated with higher odds of ICU admission compared with bronchiolitis. Children aged 1–6 months represented the largest affected age group. Furthermore, these episodes exhibited significant seasonal variation (*p* = 0.004), with higher episode volumes during winter months creating a consistent and concentrated burden on hospital resources.

The mean cohort age was 8.19 months with a male predominance (56.6%), demographics consistent with multiple previous studies [10,11]. Bronchiolitis was the predominant diagnosis (84.6%), followed by pneumonia (15.1%), aligning with findings from a Turkish cohort [11]. Conversely, studies from South Korea [12], Thailand [13], and Nigeria [14] reported pneumonia as the most common diagnosis, followed by bronchitis.

Overall, 7.1% of episodes required ICU admission, while in-hospital mortality remained low (0.08%), occurring exclusively among younger infants. Our multivariable analysis demonstrated that each additional month of age was associated with a 12% reduction in the likelihood of ICU admission, reinforcing the observation that 71% of ICU admissions occurred among infants aged 1–6 months. These findings highlight early infancy as a critical period of vulnerability to severe LRTIs. Previous studies have similarly reported that increasing pediatric age is associated with lower risks of severe bronchiolitis and pneumonia requiring intensive care [10]. Furthermore, epidemiological data from the United States demonstrated that the highest rates of severe LRTI-related hospitalization occur during the first year of life, with disease severity declining progressively with advancing age [15].

We also found that pneumonia was associated with nearly threefold higher odds of ICU admission compared with acute bronchiolitis (aOR 2.91, 95% CI 2.43–3.48, *p* < 0.001). Although bronchiolitis was the predominant diagnosis in our cohort, these findings suggest substantially greater clinical severity among children with pneumonia. The increased need for intensive care likely reflects the higher risk of respiratory compromise and the greater requirement for advanced respiratory support and close clinical monitoring. Supporting this interpretation, Alejandre et al. reported that pneumonia co-infection was frequently observed among infants admitted to the PICU with bronchiolitis, suggesting that concomitant pneumonia may substantially increase disease severity and ICU utilization [16].

Notably, KAH remained associated with higher odds of ICU admission after multivariable adjustment. This finding suggests that inter-hospital variation in ICU utilization may be influenced by factors beyond age and primary diagnosis alone and should therefore be interpreted cautiously because hospital-level clinical practices and referral patterns were not captured in the dataset. One possible explanation is the presence of unmeasured confounders, including differences in baseline comorbidity burden, referral patterns, or clinical admission practices across participating centers. Because variables such as prematurity and chronic lung disease were not available in the dataset, residual confounding cannot be excluded.

The median LOS was 4 days (IQR, 3–7) in the pediatric ward and 5 days (IQR, 3–10) in the ICU. External reports indicate highly variable hospital durations; one study reported a median PICU LOS of 2.8 days (IQR, 1.08–7.04) [13]. A systematic review of severe pneumonia cases found a median LOS of 7.9 days (IQR, 5.5–9.2) in high-income countries (HICs) and 6.4 days (IQR, 4.1–7.1) in low- and middle-income countries (LMICs) [17]. Furthermore, for very severe pneumonia requiring ICU management, the median LOS extended to 14.5 days (IQR, 10.1–18.1) in HICs and 9.2 days (IQR, 6.1–12.6) in LMICs [17].

Healthcare expenses varied significantly across hospitals, primarily because of differences in cohort volume, ICU utilization, and LOS. The cumulative direct healthcare cost for all LRTI episodes reached USD 23.0 million. Although ICU admissions represented only 7.1% of all episodes, they accounted for 45.1% of the total cost. The disproportionate ICU-related costs likely reflect the greater clinical severity observed among younger infants and patients with pneumonia, which was independently associated with higher odds of ICU admission in multivariable analysis, as well as the increased resource intensity associated with prolonged critical care management. When examining hospital-specific data, KAMC-R generated the highest Hospitalized (ICU and Ward) Episode Costs USD 10.7 million, representing 51.1% of the overall financial burden. This high total cost likely reflects KAMC-R’s status as the largest participating center. Additionally, the dataset contained extreme resource utilization outliers, which disproportionately increased total cost estimates. Sensitivity analysis excluding the upper 1% of LOS values reduced total costs substantially, while median costs remained unchanged, indicating that these high-cost cases affected overall expenditure but not the typical cost per episode.

Statistical analysis demonstrated significant seasonal variation in LRTI episodes, with higher case volumes observed during winter months. Although causative pathogens were not specifically evaluated in the present study, this pattern is consistent with previously reported seasonal circulation of respiratory viruses in Saudi Arabia, particularly respiratory syncytial virus (RSV), which typically peaks during winter and early spring [8]. A nationwide study conducted in Brazil identified clear, statistically significant seasonal patterns for LRTIs. That analysis demonstrated strong annual cyclical trends across five geographical regions, confirming that LRTIs are highly seasonal rather than uniformly distributed over time [18]. Furthermore, epidemiology data from Thailand revealed two distinct seasonal peaks of LRTIs, occurring primarily between February and March, and subsequently between July and September [13].

## 7. Limitations

Microbiological confirmation data were not consistently available for the entire cohort due to the retrospective administrative nature of the dataset. Patients were identified using ICD-10 diagnostic codes, not manual chart reviews. This electronic approach enabled a large sample size and improved generalizability. However, the risk of misclassification bias remained because of possible discrepancies in coding standards across hospitals.

The cost analysis was limited to direct medical costs based on standardized institutional rates and did not include detailed patient-level expenditures such as medications, diagnostics, or respiratory support, which may underestimate the true cost burden. Furthermore, because ED costs were fixed at USD 80 per visit, the calculated ED median costs inherently reflect this administrative ceiling. Consequently, this limitation may obscure the true resource intensity and variable costs linked to complex ED presentations.

Although the multicenter approach enhances external validity, residual variations in clinical practice protocols across participating sites may have introduced unmeasured confounding. Also, a small proportion of patients with prolonged ICU stays contributed disproportionately to total costs, which may limit the generalizability of total expenditure estimates. The episode-construction approach may have underestimated the absolute number of ED encounters because ED visits followed by hospitalization within 24 h were incorporated into the same clinical episode. Lastly, vaccination status and data on co-infection variables were not available and may have influenced disease severity and healthcare utilization.

## 8. Conclusions

In conclusion, pediatric LRTIs impose a substantial clinical and economic burden on tertiary hospitals in Saudi Arabia, particularly among infants aged 1–6 months. Although mortality remained low, prolonged hospitalizations and ICU utilization contributed disproportionately to overall healthcare expenditures. The observed winter increase in LRTI episodes and associated resource utilization highlight the importance of preventive strategies and proactive healthcare resource planning to reduce the burden of severe pediatric respiratory infections.

## Figures and Tables

**Table 1:** Demographics of pediatric LRTI patients (Patient-level dataset) N = 14,320 patients.

Characteristics		N	Frequency
Hospital	KAMC-R	8921	62.3%
	IABFH	1601	11.2%
	PMBAH	1555	10.9%
	KAH	1135	7.9%
	KAMC-J	1108	7.7%
Gender	Male	8099	56.6%
	Female	6219	43.4%
Age group	1–6 months	7079	49.4%
	7–12 months	4065	28.4%
	13–18 months	1966	13.7%
	19–24 months	1210	8.4%
Diagnosis	Acute bronchiolitis	12,118	84.6%
	Pneumonia	2166	15.1%
	Other LRTI	36	0.3%
Patient type	Emergency department	9401	65.6%
	Inpatient	4919	34.4%
In Hospital Mortality		11	0.08%

KAMC-R: King Abdulaziz Medical City—Riyadh; PMBAH: Prince Mohammed Bin Abdulaziz Hospital; KAMC-J: King Abdulaziz Medical City—Jeddah; KAH: King Abdulaziz Hospital; IABFH: Imam Abdulrahman Al Faisal Hospital.

**Table 2:** Age Group Distribution by Diagnosis.

Age Group	Acute Bronchiolitis n (%)	Pneumonia n (%)	*p*-Value
1–6 months	6631 (93.7%)	438 (6.3%)	
7–12 months	3483 (85.7%)	572 (14.3%)	
13–18 months	1380 (70.5%)	576 (29.5%)	
19–24 months	624 (51.8%)	580 (48.2%)	
Overall	—	—	<0.001

**Table 3:** Univariate and Multivariable Logistic Regression Analysis of Factors Associated with ICU Admission (N = 1007).

Variables	Univariate Analysis		Multivariate Analysis	
	OR (95% CI)	*p*-Value	aOR (95% CI)	*p*-Value
Age (months)	0.90 (0.887–0.913)	<0.001	0.88 (0.868–0.894)	<0.001
Gender				
Female	Reference	—	Reference	—
Male	1.08 (0.948–1.230)	0.246	1.06 (0.929–1.211)	0.383
Diagnosis				
Acute bronchiolitis	Reference	—	Reference	—
Pneumonia	1.56 (1.327–1.823)	<0.001	2.91 (2.429–3.483)	<0.001
Hospital Name				
IABFH	Reference	—	Reference	—
KAMC-R	0.84 (0.678–1.047)	0.123	0.82 (0.654–1.016)	0.069
KAMC-J	1.59 (1.201–2.097)	0.001	1.23 (0.922–1.643)	0.159
PMBAH	1.29 (0.990–1.690)	0.059	1.21 (0.922–1.586)	0.169
KAH	2.33 (1.795–3.013)	< 0.001	2.14 (1.644–2.788)	<0.001

OR, Odds Ratio; aOR, Adjusted Odds Ratio; CI, Confidence Interval. Logistic regression analysis was conducted using the patient-level dataset. Three patients with rare diagnoses categorized as “Other LRTI” were excluded from regression analysis because of insufficient subgroup size.

**Table 4:** Length of Stay and Cost Characteristics (episode-level dataset).

Variables	N	Median	IQR	Range
Age (months)	14,320	7	4–12	1–24
LOS in Ward (days)	6626	4	3–7	1–1102
LOS of ICU (days)	1166	5	3–10	1–1100
Ward costs (USD)	6626	907	680–1586	227–249,748
ICU Cost (USD)	1166	4333	2600–8665	867–953,184

USD: United States Dollar. ICU LOS and ICU cost analyses were performed using the episode-level dataset; therefore, repeated ICU episodes from the same patient could contribute more than once.

**Table 5:** Hospitalized (ICU and Ward) Episode Costs Stratified by Hospital.

Hospital	N	Total Cost (USD)	Median Cost (IQR), (USD)
KAMC-R	3722 (56.2%)	10,796,080 (51.1%)	680 (453–1587)
KAMC-J	755 (11.4%)	2,311,107 (10.9%)	907 (680–2040)
PMBAH	856 (12.9%)	4,217,547 (20.0%)	1133 (680–2267)
KAH	806 (12.2%)	2,398,200 (11.4%)	907 (680–2000)
IABFH	487 (7.3%)	1,403,187 (6.6%)	1320 (680–2947)
Total	6626 (100%)	21,126,120 (100%)	907 (680–1813)

## Data Availability

The data that support the findings of this study are not publicly available due to institutional restrictions and patient confidentiality but may be available from the corresponding author upon reasonable request.

## Supplementary Materials

The following supporting information can be downloaded at https://www.mdpi.com/article/10.3390/pediatric18030071/s1, Table S1: ICD-10 codes used for lower respiratory tract infection identification.
