# Supplementary material for: Healthcare Utilization and Economic Burden of Pediatric Lower Respiratory Tract Infections Across Five Tertiary Hospitals in Saudi Arabia
Source: Pediatr Rep. 2026 May 25;18(3):71. doi: 10.3390/pediatric18030071 (PMC13305304; doi:10.3390/pediatric18030071)
Supplement: Supplementary file 1 [file pediatrrep-18-00071-s001.zip › pediatrrep-4303816-supplementary.pdf]

Table S1: ICD-10 Codes Used for Lower Respiratory Tract Infection Identification

Code	Diagnosis
J20	Acute bronchitis
J20.0	Acute bronchitis due to Mycoplasma pneumoniae
J20.1	Acute bronchitis due to Haemophilus influenzae
J20.2	Acute bronchitis due to Streptococcus
J20.3	Acute bronchitis due to Coxsackievirus
J20.4	Acute bronchitis due to Parainfluenza virus
J20.5	Acute bronchitis due to Respiratory syncytial virus (RSV)
J20.6	Acute bronchitis due to Rhinovirus
J20.7	Acute bronchitis due to Echovirus
J20.8	Acute bronchitis due to other specified organisms
J20.9	Acute bronchitis, unspecified
J21	Acute bronchiolitis
J21.0	Acute bronchiolitis due to RSV
J21.1	Acute bronchiolitis due to Human metapneumovirus
J21.8	Acute bronchiolitis due to other specified organisms
J21.9	Acute bronchiolitis, unspecified
J22	Unspecified acute lower respiratory infection (NOS)
J12.0	Adenoviral pneumonia
J12.1	RSV pneumonia
J12.2	Parainfluenza virus pneumonia
J12.3	Human metapneumovirus pneumonia
J12.8	Other viral pneumonia
J12.9	Viral pneumonia, unspecified
J13	Streptococcus pneumoniae pneumonia
J14	Haemophilus influenzae pneumonia
J15.0–J15.9	Bacterial pneumonia due to various organisms
J16.0	Chlamydial pneumonia
J16.8	Other specified infectious pneumonia
J17.0	Pneumonia in diseases classified elsewhere
J18.0–J18.9	Pneumonia, unspecified types
B97.4	Respiratory syncytial virus as the cause of diseases classified to other chapters
